# Remote person-centred care and long-term medication management in primary care: post hoc analysis of a randomised controlled trial

**DOI:** 10.1136/bmjopen-2026-118720

**Published:** 2026-07-01

**Authors:** Stina Mannheimer, Andreas Fors, Anna Holst, Hanna Gyllensten

**Affiliations:** 1 Institute of Health and Care Sciences, Sahlgrenska Academy, University of Gothenburg, Gothenburg, Sweden; 2 Centre for Person-Centred Care (GPCC), Sahlgrenska Academy, University of Gothenburg, Gothenburg, Sweden; 3 Region Västra Götaland, Research, Education, Development and Innovation, Primary Health Care, Gothenburg, Sweden; 4 School of Public Health and Community Medicine, Institute of Medicine, University of Gothenburg, Gothenburg, Sweden

**Keywords:** Primary Care, Polypharmacy, Multimorbidity, Person-Centered Care

## Abstract

**Objectives:**

To explore refill adherence to prescribed treatment for chronic heart failure (CHF) and/or chronic obstructive pulmonary disease (COPD), during and after a remote person-centred care (PCC) intervention.

**Design:**

Post hoc analysis of a randomised controlled trial cohort.

**Setting:**

We used data from the PROTECT (Person-Centred Care at Distance) randomised controlled trial in Swedish primary care, which were linked to data from the Swedish Prescribed Drug Register.

**Participants:**

A total of 222 participants from nine primary care centres were enrolled in PROTECT. This study included participants with dispensed medications for CHF and/or COPD, excluding those with multidose dispensing (intervention n=50; control n=49, 42% women, mean age 69 years).

**Interventions:**

PROTECT evaluated a PCC intervention using a digital platform and structured telephone support in addition to usual care versus usual care alone.

**Outcome measures:**

Refill adherence was compared between groups, defining appropriate medication supply as ≥80% of days covered over 2 years. We also examined associations between refill adherence and number of long-term conditions and medications.

**Results:**

Refill adherence did not differ significantly between groups. A higher number of long-term conditions was associated with lower odds of refill adherence (adjusted OR (aOR) per additional condition: 0.71; 95% CI 0.50 to 0.97; p=0.041), as were more long-term medications (aOR per additional medication class: 0.84; 95% CI 0.71 to 0.98; p=0.029).

**Conclusions:**

The PROTECT intervention did not improve refill adherence, suggesting that future person-centred interventions may require more structured medication-management focus. Increasing numbers of long-term conditions and medications were both associated with lower refill adherence.

**Trial registration number:**

NCT03183817.

**STRENGTHS AND LIMITATIONS OF THIS STUDY**

- Robust data sources: Complete registry data were combined with a well-defined population from a clinical randomised controlled trial, ensuring internal validity.
- Clinical relevance: Trial linkage ensured participants were clinically stable at baseline and had appropriate indications for treatment, enhancing relevance to primary care.
- Low financial barriers: Sweden’s national reimbursement scheme minimises cost-related non-adherence.
- Limited power and intervention focus: High baseline adherence, small sample size and an intervention not specifically targeting medication-taking likely limited detectable effects; analyses should be considered exploratory.
- Measurement and contamination limitations: Refill adherence based on dispensing cannot confirm actual consumption, and person-centred elements in usual care may have diluted intervention effects.

## Introduction

 Chronic heart failure (CHF) and chronic obstructive pulmonary disease (COPD) commonly contribute to multimorbidity (defined as ≥2 long-term conditions) and generate prescriptions of multiple medications.^1 2^ Challenges associated with long-term medication^3^ may reduce patients’ ability or willingness to refill prescriptions frequently enough to enable full therapeutic effect. In other words, these challenges can lead to suboptimal refill adherence, which might be associated with compromised health outcomes^4 5^ and increased healthcare costs.^6^ Several meta-analyses suggest that behavioural interventions may be effective in supporting long-term medication use among older adults prescribed multiple medications.^7 8^ Person-centred care (PCC) brings together the patient’s perspective with medical expertise in a partnership that highlights the importance of acknowledging the patient as a person^9^ and strives to enhance the patient’s self-efficacy^10^; that is, their confidence in their ability to manage health conditions and to solve related challenges. Through strengthened patient–clinician partnership and increased attention to individual barriers and resources, PCC may also indirectly influence patients’ ability to manage long-term medication use. Previous research has suggested that nurse-led support and trustful patient–clinician relationships may support medication adherence in chronic disease management, including among patients with cardiovascular disease and multimorbidity.^11 12^

The effects of PCC, which are core in the ongoing transition to person-centred and integrated care in Sweden,^13^ have been highlighted in several studies.^14 15^ Data for the present study were drawn from one of these trials: the randomised controlled trial PROTECT (Person-Centred Care at Distance), in which a remote PCC intervention was conducted in a primary care population.^16^ The intervention, based on a PCC framework developed by the University of Gothenburg Centre for Person-Centred Care,^10^ demonstrated a short-term improvement in patients’ perceptions of self-efficacy,^16^ and resulted in both lower societal costs and a slight improvement in quality-adjusted life-years among those receiving the PCC add-on intervention compared with usual care.^17^ Whether the intervention could also have influenced long-term medication-taking remained unclear.

The primary aim of the present study was to explore refill adherence to prescribed treatment for CHF and/or COPD, during and after the remote PCC intervention in the PROTECT trial. Additionally, to examine associations between refill adherence and number of long-term conditions and medications, respectively.

## Materials and methods

### Study design

This study reports results from an exploratory post hoc analysis of the PROTECT randomised controlled trial evaluating remote PCC,^16 18^ examining refill adherence as an outcome. The PROTECT trial reported no significant between-group differences in the primary outcome: a composite score of changes in general self-efficacy, hospitalisation and death at the 6-month follow-up. However, the per-protocol analysis revealed a significant improvement in the intervention group at the 3-month follow-up,^16^ driven by an improvement in self-reported general self-efficacy. Detailed descriptions of the study methods and procedures have been published previously,^16 18^ and the published study protocol is also provided as online supplemental file 1. A brief overview is given below.

### Population and setting

PROTECT was conducted during 2018–2020 with patients (n=222) recruited from nine primary healthcare centres in Gothenburg, Sweden. Inclusion criteria were a diagnosis of CHF (International Classification of Diseases (ICD)-10 codes: I50.0–I50.9) and/or COPD (ICD-10 codes: J43.0, J44.0–J44.9), being registered with one of the participating primary healthcare providers, being able to communicate in Swedish, and having access to a device with internet connection. Exclusion criteria were impairments that could interfere with follow-up or prevent the person from using eHealth support, no registered address, expected survival of <12 months, ongoing documented diagnosis of alcohol or drug abuse, or participation in a conflicting study. Randomisation, based on a computer-generated list created by a third party and stratified by age (<65 or ≥65 years) and diagnostic group (COPD, CHF or both), allocated 112 participants to the control group (mean age: 70.4, 46% women) and 110 participants to the intervention group (mean age: 71.1, 46% women).^16^

In the current post hoc study, exclusion criteria applied to the PROTECT participants were: (1) receiving medication via multidose dispensing, as data in the Swedish Prescribed Drug Register are inconsistent for this group, (2) having no dispensed medication for CHF and/or COPD during the 2 years following inclusion in PROTECT and (3) having dispensed prescriptions of medication for CHF and/or COPD that did not allow for calculation of daily dose. All analyses were conducted on the remaining participants.

### Intervention group

In addition to usual care, participants in the intervention group received remote PCC over a 6-month period, combining telephone support and access to a digital platform. The frequency, timing and extent of contacts were tailored according to each participant’s needs and preferences and reflected the ongoing PCC partnership throughout the intervention period. The support was provided by a team of five designated healthcare professionals trained in PCC. Participants often had repeated contact with the same healthcare professional, although support could also be provided by different members of the PCC team during the intervention period. The healthcare professionals’ work was grounded in a framework developed by the University of Gothenburg Centre for Person-Centred Care^10^ that emphasises building on the patient’s narrative to identify resources, needs, possibilities and potential barriers. The conversations were open (ie, not guided by a predefined manual) and could cover topics such as symptoms, treatment, quality of life, ability to participate in activities and social networks. Although many participants brought up medication management, it was not addressed in a standardised manner. The conversations concluded with the co-creation of personal health plans, integrating the patient’s perspectives and goals with the healthcare professional’s expertise. The plan was uploaded to the digital platform and was considered and revised during follow-up conversations. The platform enabled ongoing dialogue, allowing patients to communicate with the team of healthcare professionals providing the intervention, review and update their health plan, track symptoms visually, invite support persons and access relevant online resources.^16 18^

### Control group

Participants in the control group received usual care, and medication adjustments were managed according to the physician’s assessment and based on current guidelines.^19 20^ The control group were informed of group allocation per telephone but had no follow-up conversations.

### Data collection and preparation

Data on dispensed medications were obtained during 2024 from the Swedish Prescribed Drug Register (held by the National Board of Health and Welfare) and linked to study participants. Linkage was performed by Statistics Sweden (SCB) using the Swedish personal identity number in accordance with ethical approval and Swedish data-protection regulations. The dataset provided to the research team was pseudonymised and contained only study-specific identifiers; no personal identity numbers were available to the researchers. We included all dispensing within 2 years after the individual study inclusion date (ie, 730 days). The following variables were assessed:

1. Anatomical Therapeutic Chemical (ATC) codes and item numbers of dispensed medication.
2. Dates of prescription and dispensing.
3. Dosage text.
4. Number of packages and number of tablets/units per dispensed package.

The following key medication categories used for treating CHF and/or COPD were extracted from the Swedish Prescribed Drug Register to assess refill adherence: ACE inhibitors/angiotensin II receptor blockers (ACEi/ARB, ATC code: C09), beta blockers (ATC code: C07), mineralocorticoid receptor antagonists (MRA, ATC code: C03D), sodium-glucose cotransporter 2 inhibitors (SGLT2 inhibitors, ATC code: A10BK), any kind of inhaler (ATC code: R03) and roflumilast (ATC code: R03DX07). Diuretics were not included because dosing is frequently adjusted according to symptoms and clinical status, making refill coverage difficult to interpret as non-adherence. To capture each participant’s long-term medication use, we included all ATC classes typically representing long-term therapy (listed in online supplemental file 2, appendix p.3). Each unique ATC class was defined by the first three characters of the code and counted once per participant regardless of how many times a medication was dispensed. The number of unique long-term medications per participant was used as a continuous variable in the statistical analyses.

Demographic characteristics and diagnoses were retrieved from primary care electronic health records via PROTECT data. To select representative diagnoses for assessment of each participant’s total number of long-term conditions during the study period, we used the Charlson Comorbidity Index adapted for ICD-10 codes,^21^ a validated tool for assessment of burden of chronic disease. To ensure clinical relevance for our primary care population, we screened all diagnoses in our dataset against the index list and subsequently added five long-term conditions that were prevalent in our primary care population: depression, anxiety, chronic pain, osteoarthritis and hypertension. The full list of included diagnoses is presented in online supplemental file 2, appendix p.4

### Statistical analyses

Descriptive statistics were used to characterise the participants. Categorical variables were presented as counts and compared between groups using a χ² test or Fisher’s exact test (if expected cell counts were <5). Age, the only continuous variable, was presented as mean±SD and compared between groups using the independent samples t-test. The proportion of participants with dispensing within each medication category (therapy presence) was compared between groups using a χ^2^ test or Fisher’s exact test.

Refill adherence^22^ was estimated for each of the participants’ medications by relating the number of doses dispensed and the dispensing interval to the demand based on prescribed daily dose. If prescribed dosage changed over time, the estimated expected consumption was adjusted accordingly using the dosage text available at each dispensing. This indicated each participant’s access to medication during the study period, referred to as the proportion of days covered. An appropriate supply was defined as coverage of ≥80% of days during the 2-year study period (ie, ≥584 of 730 days).^23^ Refill adherence for CHF-related and COPD-related medications was compared between the intervention and control groups using χ² test or Fisher’s exact test. A two-sided p<0.05 was considered statistically significant.

For the secondary aim, refill adherence was treated as a binary variable (adherent vs non-adherent), with adherent defined as ≥80% coverage for all of each participant’s medications within the assessed medication categories. Consequently, participants were classified as non-adherent if coverage was <80% for any medication with an active prescription. Unadjusted numbers of long-term conditions and medication classes were compared between adherent and non-adherent participants using Wilcoxon rank-sum tests. These analyses were complemented by multivariable logistic regression models adjusted for age, sex, smoking status (current, previous, never), education (higher vs up to secondary) and civil status (cohabiting vs living alone). Refill adherence served as the outcome, with (1) the number of long-term conditions and (2) the number of long-term medication classes examined in separate models. ORs with 95% CIs were reported. A two-sided p<0.05 was considered statistically significant. These analyses were conducted in the combined population (intervention and control groups together), as a register-based observational analysis of the trial cohort.

StataNow V.18.5 SE (StataCorp) was used to calculate refill adherence. All other statistical computing and graphics were performed in R V.4.5.0 (R Core Team, 2025).

### Patient and public involvement

Patient representatives were involved in the design of the original PROTECT randomised controlled trial.^16^ In addition, participating patients contributed through person-centred consultations that informed the intervention. Patients and the public were not involved in the design, analysis, or reporting of this post hoc register-linked study.

### Reporting guidelines

The current study is reported according to the Consolidated Standards of Reporting Trials (CONSORT) guidelines^24^; the completed checklist is provided in online supplemental file 3, with further details referenced in the CONSORT e-supplement (online supplemental file 4).

## Results

**Figure 1:**
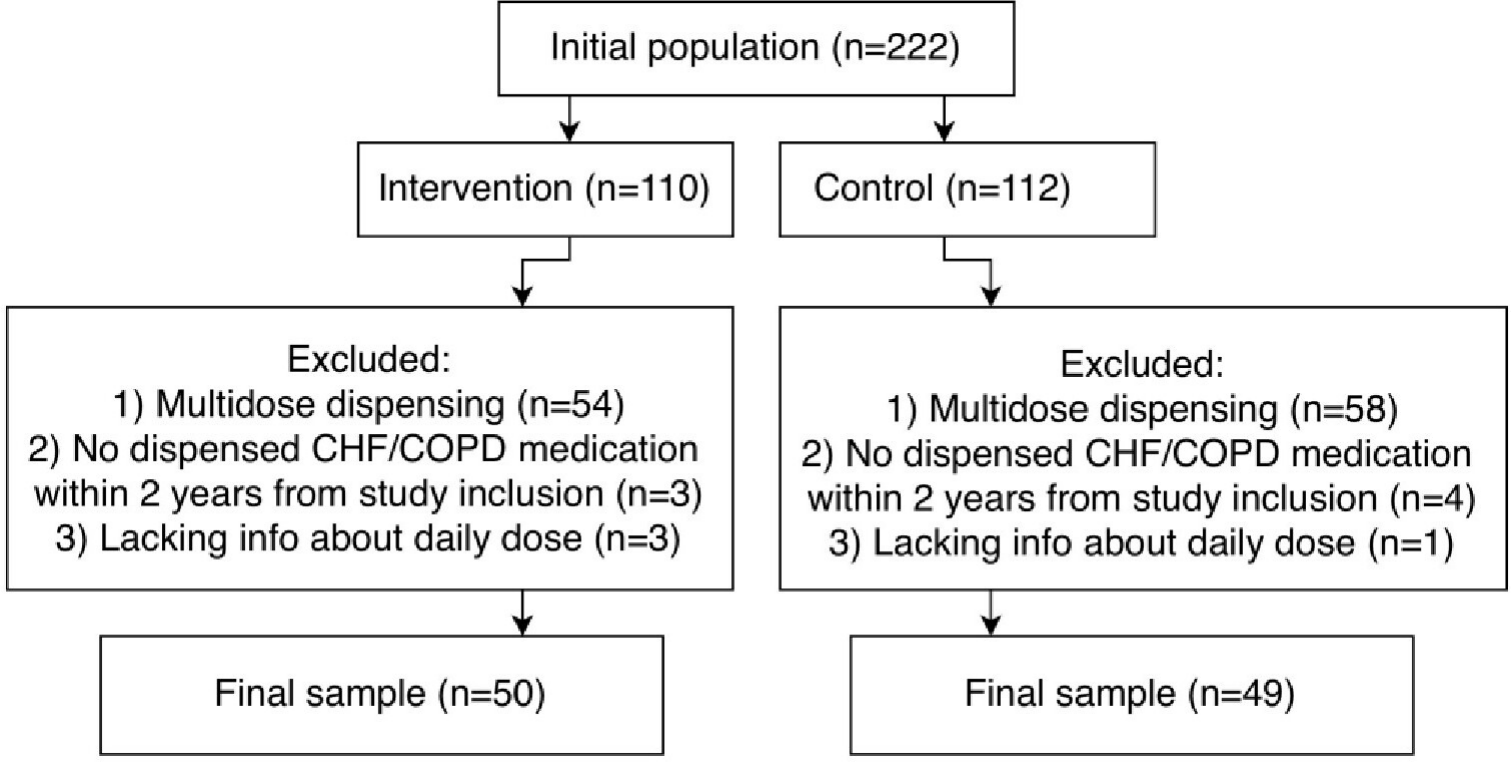
Flow chart of participant selection. CHF, chronic heart failure; COPD, chronic obstructive pulmonary disease.

Of the 222 PROTECT participants, 123 were excluded from the present post hoc analysis. The main reasons were multidose dispensing, absence of dispensed CHF/COPD medication during follow-up, or dispensing records that did not allow calculation of daily dose. These exclusions were well balanced between the intervention and control groups across exclusion criteria, resulting in a final analysis population of 50 intervention and 49 control participants (figure 1). Measured baseline characteristics, the distribution of diagnoses (CHF, COPD or both), and the prevalences of the most common comorbidities were all similar between the groups (table 1). The median number of long-term medications from unique ATC groups dispensed to the participants during 2 years from study inclusion was six in both groups. Of the medications used for treatment of CHF and COPD, SGLT2 inhibitors were only prescribed for treatment of diabetes, and roflumilast was not dispensed frequently enough for assessment of refill adherence. We therefore focused on four key medication categories: ACEi/ARB, beta blockers, MRA and inhalers. In the combined population (n=99), ACEi/ARB was the most common therapy (n=66), followed by inhalers (n=60), beta blockers (n=59) and MRA (n=19). The number of participants with active treatment from each of these four key medication categories did not differ significantly between the groups (smallest p=0.19).

**Table 1:** Baseline participant characteristics

Characteristics	Control (n=49)	Intervention (n=50)	P value
Age, mean (SD)	68.5 (8.7)	69.8 (9.0)	0.48
Women, n (%)	21 (42.9)	21 (42.0)	0.93
Medications			
ACEi/ARB, n	33	33	0.89
MRA, n	12	7	0.19
Beta blockers, n	26	33	0.19
Inhalers, n	28	32	0.49
Number of medications*, median (IQR)	6 (4)	6 (3.75)	0.28
Diagnosis			
CHF	17	15	0.67
COPD	28	28	1.0
CHF and COPD	4	7	0.52
Comorbidities			
Hypertension	38	40	1.0
Atrial fibrillation	18	20	0.95
Chronic pain including osteoarthritis	16	18	0.94
Coronary artery disease	11	16	0.44
Depression and/or anxiety	9	14	0.40
Cancer	11	13	0.90
Diabetes	8	11	0.68
Kidney disease	5	5	1.0
Previous stroke/TIA	6	2	0.16

* Long-term medications from different Anatomical Therapeutic Chemical groups.

ACEi, ACE inhibitor; ARB, angiotensin II receptor blocker; CHF, chronic heart failure; COPD, chronic obstructive pulmonary disease; MRA, mineralocorticoid receptor antagonist; TIA, transient ischaemic attack.

The number of participants per group with dispensing from any of the four assessed medication classes (therapy presence) is displayed in table 2 along with the number and proportion of participants with refill adherence ≥80%. In both groups, over 80% of patients with ACEi/ARB and over 75% of patients with beta blockers showed this level of adherence, with no significant between-group differences (p=0.74 and 0.86, respectively). Therapy with MRA was dispensed to few participants, and no significant group differences were seen regarding refill adherence (58.3% vs 28.6%; p=0.35). For inhalers, the proportion of participants with refill adherence of at least 80% was comparatively lower than in the other medication categories (50.0% in control vs 56.2% in intervention; p=0.63). In summary, across all four medication categories, no statistically significant differences regarding refill adherence were observed between intervention and control participants.

**Table 2:** Therapy presence and proportion with refill adherence ≥80% (ie, proportion of days covered with medication supply during the 2-year follow-up), by randomisation group

Treatment	Control	Intervention	OR (95% CI)	P value
ACEi/ARB				
Therapy presence, n	33	33	0.94 (0.41 to 2.17)	0.89
Refill adherence ≥80%, N (N/n)	27 (81.8%)	28 (84.8%)	1.24 (0.34 to 4.56)	0.74
Beta blockers				
Therapy presence, n	26	33	1.72 (0.76 to 3.86)	0.19
Refill adherence ≥80%, n (n/N)	20 (76.9%)	26 (78.8%)	1.11 (0.32 to 3.84)	0.86
MRA				
Therapy presence, n	12	7	0.50 (0.18 to 1.41)	0.19
Refill adherence ≥80%, n (n/N)	7 (58.3%)	2 (28.6%)	0.29 (0.04 to 2.11)	0.35*
Inhalers				
Therapy presence, n	28	32	1.33 (0.59 to 2.99)	0.49
Refill adherence ≥80%, n (n/N)	14 (50.0%)	18 (56.2%)	1.29 (0.46 to 3.56)	0.63

ORs are given for intervention versus control. P values were calculated using a χ2 test except where indicated.

* Fisher’s test used due to cell counts <5.

ACEi, ACE inhibitors; ARB, angiotensin II receptor blockers; MRA, mineralocorticoid receptor antagonist.

**Figure 2:**
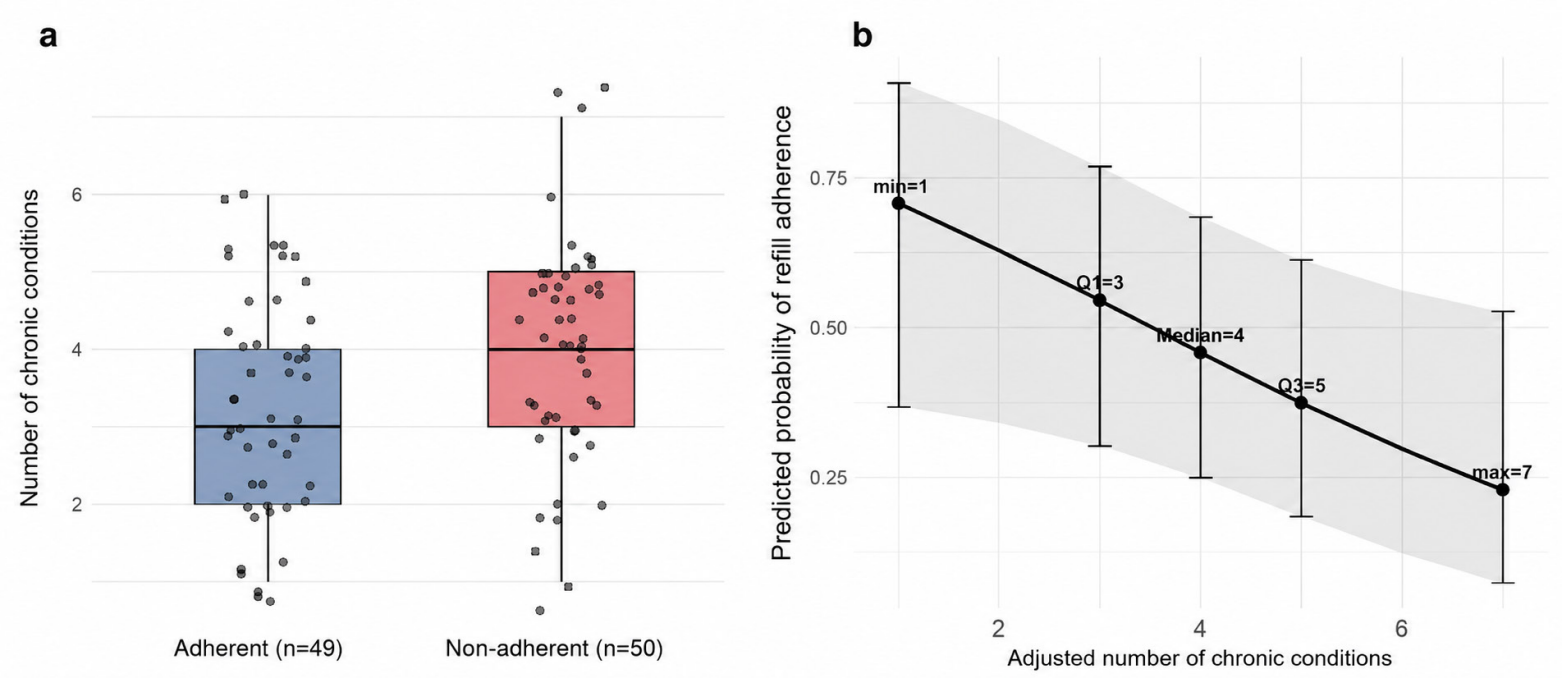
(a) Number of long-term conditions by refill adherence group: adherent median (IQR): 3 (2–4) versus non-adherent: 4 (3–5); Wilcoxon rank-sum p=0.023. Points indicate individual participants. (b) Predicted probability of refill adherence to medications for CHF and/or COPD, based on a multivariable logistic regression adjusted for age, sex, smoking status, education and civil status. Each additional long-term condition was associated with lower odds of refill adherence (adjusted OR 0.71; 95% CI 0.50 to 0.97; p=0.041). The line shows adjusted predictions with 95% CIs. Points indicate the minimum, Q1, median, Q3 and maximum observed number of long-term conditions. CHF, chronic heart failure; COPD, chronic obstructive pulmonary disease.

Assessing the combined population regarding the binary refill adherence variable (defined in the Methods section) resulted in 49 participants classified as adherent and 50 as non-adherent. The number of chronic conditions among participants differed significantly between these two groups: participants classified as adherent had a median of 3 long-term conditions (IQR 2–4) compared with 4 among non-adherent participants (IQR 3–5; Wilcoxon rank-sum p=0.023; figure 2a). In the adjusted logistic regression model, each additional long-term condition was associated with lower odds of refill adherence to CHF and/or COPD medication (adjusted OR per additional condition: 0.71; 95% CI 0.50 to 0.97; p=0.041). Consistently, the predicted probability of refill adherence decreased as the number of chronic conditions increased (figure 2b).

**Figure 3:**
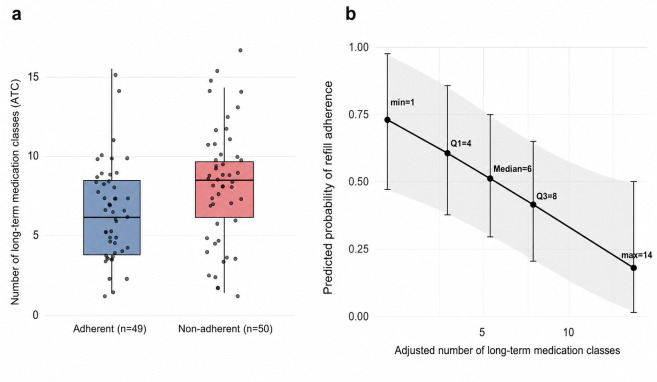
(a) Number of long-term medication classes (ATC) by refill adherence group: adherent median (IQR): 5 (3–7) versus non-adherent: 7 (5–8); Wilcoxon rank-sum p=0.006. Points indicate individual participants. (b) Predicted probability of refill adherence to medications for CHF and/or COPD, based on a multivariable logistic regression adjusted for age, sex, smoking status, education and civil status. Each additional long-term medication class was associated with lower odds of refill adherence (adjusted OR 0.84; 95% CI 0.71 to 0.98; p=0.029). The line shows adjusted predictions with 95% CIs. Points indicate the minimum, Q1, median, Q3 and maximum observed number of medication classes. CHF, chronic heart failure; COPD, chronic obstructive pulmonary disease.

Further, participants classified as adherent were dispensed a significantly lower median number of long-term medications compared with those classified as non-adherent (adherent median (IQR): 5 (3-7) vs non-adherent: 7 (5-8); Wilcoxon rank-sum p=0.006; figure 3a). In the adjusted logistic regression model, each additional long-term medication class was associated with lower odds of refill adherence to medication for CHF and/or COPD (adjusted OR per additional medication class: 0.84; 95% CI 0.71 to 0.98; p=0.029; figure 3b).

None of the demographic or socioeconomic covariates were significantly associated with refill adherence in the adjusted models. Smoking status showed a pattern of higher refill adherence among current smokers across both models, although CIs were wide, and no significant association was observed for former smokers.

## Discussion

### Summary

Our study found no significant difference in refill adherence to prescribed medication for CHF and/or COPD between the intervention and control groups. In the combined population, increasing multimorbidity and number of long-term medications were both associated with a stepwise decline in refill adherence to medication for CHF and/or COPD.

### Strengths and limitations

An important strength of this study is the use of complete registry data combined with a well-defined study population derived from a randomised controlled trial. Linkage to the clinical trial ensured that participants were clinically stable at baseline (ie, not hospitalised), and had relevant conditions justifying treatment, enhancing relevance to primary care. In comparison, fully register-based studies often lack information on clinical appropriateness and must infer treatment eligibility from dispensing data alone. Another strength of conducting this study within Swedish primary healthcare is that financial barriers to treatment are small, as all included prescription medicines are covered by the national reimbursement scheme, with patient co-payments capped at approximately 130 EUR per year.^25^ However, this system may incentivise medication stockpiling, and we therefore accounted for potential oversupply in the analyses.

The study also has several limitations. The person-centred intervention in PROTECT was not specifically designed to target medicine-taking but rather was based on patients’ narratives and the issues raised during the conversations. It is possible that the lack of effect of the intervention reflects the high refill adherence observed in both groups at baseline, leaving limited margins for improvement, together with the relatively small study population. The sample size in PROTECT was based on the composite score constituting the primary trial outcome and the study was not specifically powered for refill adherence analyses. In addition, this post hoc analysis was not prespecified, and the intervention did not include a standardised medication-management component, unlike several previous studies specifically targeting medication use. Findings should therefore be interpreted as exploratory rather than confirmatory. Previous analyses from the full PROTECT trial population did not demonstrate associations between contact frequency and the main trial outcome.^26^ Given the substantially smaller size of the present post hoc cohort, additional analyses of contact frequency and refill adherence were considered unlikely to provide robust or clinically informative results. Moreover, because the control group was not standardised regarding the use of PCC, elements of PCC may have occurred, potentially diluting or enhancing the effect of the intervention. In addition, because no medication review or chart review was performed, treatment discontinuation could not be distinguished from non-adherence in the dispensing data. Finally, all dispensing-based measures of refill adherence have inherent limitations, since actual medication consumption and the reasons for non-adherence cannot be fully determined.

### Comparison with existing literature

Our study did not demonstrate an effect of the PCC intervention on refill adherence. However, a previous study evaluating an intervention specifically targeting medication management in older adults with multimorbidity found that medication reviews improved pharmacological parameters related to adverse drug events, with the greatest benefits observed among those with frailty.^27^ In addition, cost-effectiveness has been demonstrated for a PCC model that included pharmacist-led medication reviews and a focus on collaborative health goal-setting and patient empowerment among older adults with multimorbidity in primary care.^28^ Aligning with our study, previous research in primary healthcare has likewise shown associations between declining refill adherence and advanced multimorbidity or frailty.^29^ Our study contributes to this evidence base and indicates that polypharmacy may also be associated with difficulties in sustaining optimal medicine-taking.

### Implications for research and practice

It is less certain whether remote and more general interventions like PROTECT can be expected to influence medication use. Further research is required to explore whether and how PCC interventions targeting medication management can support patients in managing multiple medications and maintaining quality of life.^28^ Our findings can inspire the design of future studies exploring medication-taking, which could benefit from recognising the number of comorbidities and/or medications as stratification variables.

It is important in this context to reflect on the implication of the term ‘adherence’. Poor refill adherence to prescribed long-term medication, regardless of cause, may indicate a weak therapeutic alliance between the patient and clinician, characterised by inadequate information, low trust, or lack of shared decision-making. Previous research highlights that a large proportion of older adults with multimorbidity and multiple medications find medication management challenging.^3^ Further, it is well known that patients with multimorbidity are at higher risk of adverse events from polypharmacy, which adds to the complexity of care for these patients. It must be remembered that while each treatment recommended by an evidence-based single-condition guideline might be rational, the combination of recommendations for a patient with multimorbidity can be unrealistic, counterproductive, or even harmful. To improve care for patients with commonly occurring multimorbidity, the European Patients’ Forum has called for patient empowerment,^30^ and the National Institute for Health and Care Excellence guidelines recommend a holistic approach where quality of life is central and patients’ preferences and situations are carefully elicited.^31^ To maximise benefits and minimise harms associated with medication, existing evidence suggests that healthcare should strive for person-centred prescribing practices, in which medication-related issues are openly addressed within a collaborative patient-clinician partnership.^32^ Further, medication-taking should be optimised by prioritising essential treatments and applying PCC that integrates patients’ perspectives, resources, and preferences with clinicians’ medical expertise.^33^ Patients can then be supported to make appropriate decisions regarding the management of their own health, including medicine-taking.^34^

## Conclusions

We found no support that the PROTECT intervention was associated with improved refill adherence to medication for CHF and/or COPD. For trial participants, increasing numbers of long-term conditions and medications were both associated with a stepwise decline in refill adherence to medication for CHF and/or COPD. These findings suggest an association between multimorbidity, polypharmacy and refill adherence in primary care.

## Supplementary material

10.1136/bmjopen-2026-118720 online supplemental file 1

10.1136/bmjopen-2026-118720 online supplemental file 2

10.1136/bmjopen-2026-118720 online supplemental file 3

10.1136/bmjopen-2026-118720 online supplemental file 4

## Data Availability

Data may be obtained from a third party and are not publicly available.
