# Supplementary material for: Remote person-centred care and long-term medication management in primary care: post hoc analysis of a randomised controlled trial
Source: BMJ Open. 2026 Jul 1;16(7):e118720. doi: 10.1136/bmjopen-2026-118720 (PMC13331147; doi:10.1136/bmjopen-2026-118720)
Supplement: online supplemental file 2 [file bmjopen-16-7-s002.docx]

**Supplementary appendix**

Supplement to: Mannheimer S, Fors A, Holst A, Gyllensten H. **Remote person-centred care and long-term medication management in primary care:** post-hoc analysis of a randomised controlled trial

**APPENDIX**

Effects of a person-centred remote intervention on long-term medication management: post-hoc analysis of a trial population

Stina Mannheimer^1,2,*^, Andreas Fors^1,2,3^, Anna Holst^4^, Hanna Gyllensten^1,2^

1. Institute of Health and Care Sciences, Sahlgrenska Academy, University of Gothenburg, Gothenburg, Sweden.

2. University of Gothenburg Centre for Person-Centred Care (GPCC), Sahlgrenska Academy, University of Gothenburg, Gothenburg, Sweden.

3. Region Västra Götaland, Research, Education, Development and Innovation, Primary Health Care, Gothenburg, Sweden.

4. School of Public Health and Community Medicine, Institute of Medicine, University of Gothenburg, Gothenburg, Sweden.

Corresponding author

Stina Mannheimer, Medical Doctor, PhD-student

Institute of Health and Care Sciences, Gothenburg University

Arvid Wallgrens backe hus 2

Box 457

40530 Gothenburg

+46 730 250868

<mailto:stina.mannheimer@gu.se>

**Supplement 1:** Medication categories used to calculate number of long-term medications.

| **Medication category** | **Anatomical Therapeutic Chemical  (ATC) group** |
| --- | --- |
| Agents acting on the renin–angiotensin system | C09 |
| Analgesics (paracetamol, opioids, combination analgesics) | N02 |
| Anti-dementia drugs | N06D |
| Antidepressants | N06A |
| Anti-diabetics | A10 |
| Antigout preparations | M04A |
| Anti-inflammatory drugs | M01 |
| Antithrombotic agents | B01A |
| Beta-blocking agents | C07 |
| Cardiac therapy (e.g., antiarrhythmics, cardiac glycosides) | C01 |
| Calcium channel blockers | C08 |
| Diuretics | C03 |
| Drugs for acid-related disorders | A02 |
| Drugs for bone diseases | M05 |
| Drugs for obstructive airway diseases | R03 |
| Drugs used in benign prostatic hypertrophy | G04C |
| Lipid-modifying agents | C10 |
| Neuroleptics, anxiolytics, sedatives | N05 |
| Other antiepileptics (gabapentin, pregabalin—commonly for chronic pain) | N03AX |
| Thyroid therapy | H03 |

**Supplement 2:** Diagnoses used to calculate number of long-term conditions.

| **Diagnose category** | **ATC codes** |
| --- | --- |
| **Anxiety disorders** | F40–F41 |
| **Atrial fibrillation/flutter** | I48 |
| **Cerebrovascular disease** | I63–I64, G45 |
| **Chronic kidney disease** | N18–N19 |
| **Chronic pain** | R52.1–R52.2 |
| **Chronic pulmonary disease** | I27, J40-J47 |
| **Congestive heart failure** | I50 |
| **Dementia** | F00–F03, G30 |
| **Depression** | F32–F33 |
| **Diabetes mellitus** | E10–E14 |
| **Hypertension** | I10–I15 |
| **Ischaemic heart disease** | I20–I25 |
| **Malignancy** | C00–C97 |
| Mild Liver Disease | K70-K71, K73-K74, K76 |
| **Osteoarthritis** | M15–M19 |
| Peptic Ulcer Disease | K25-K27 |
| Rheumatic Disease | M05-M06 |

*Diagnoses were selected based on the Charlson Comorbidity Index adapted for ICD-10 codes, and on relevance in the current primary healthcare population.*
