# Supplementary material for: Remote person-centred care and long-term medication management in primary care: post hoc analysis of a randomised controlled trial
Source: BMJ Open. 2026 Jul 1;16(7):e118720. doi: 10.1136/bmjopen-2026-118720 (PMC13331147; doi:10.1136/bmjopen-2026-118720)
Supplement: online supplemental file 4 [file bmjopen-16-7-s004.docx]

**E-supplement**

**Description of the Original Trial Design**

This supplementary file provides information on the original trial design to support CONSORT reporting for the present post-hoc analysis.

7. Objectives

The original trial aimed to evaluate the effect of person-centred care, delivered through a combined digital platform and structured telephone support, on patient-relevant benefits including general self-efficacy and clinical outcomes.

Benefits were assessed through a composite primary endpoint capturing changes in general self-efficacy and the occurrence of hospitalisation or death. Potential harms, including deterioration in self-efficacy, hospitalisation, and death, were monitored as part of this composite outcome and follow-up assessments.

9. Trial Design

The original study was a multicentre, parallel-group randomised controlled trial with a 1:1 allocation ratio. Participants were randomised to either person-centred care in addition to usual care or usual care alone. The trial was designed within a superiority framework to evaluate whether the intervention improved patient-relevant outcomes compared with usual care.

10. Changes to trial protocol

No important changes were made to the trial design, eligibility criteria, outcomes, or planned analyses after trial commencement. Both intention-to-treat and per-protocol analyses were prespecified in the study protocol.

[14. Outcomes](https:/resources.equator-network.org/reporting-guidelines/consort/items/outcomes.html?utm_source=consort&utm_medium=checklist&utm_campaign=CONSORT_2025_v1_1)

The prespecified primary outcome was a composite of change in general self-efficacy, hospitalisation due to COPD or CHF, and death at 6 months after randomisation. Secondary outcomes included healthcare utilisation, general self-efficacy, health-related quality of life, anxiety and depression, disease-specific symptom measures, and health economic outcomes, as described in the published protocol.

15. Harms

Harms were defined as hospitalisation due to COPD or CHF and all-cause death. These outcomes were prospectively collected during the 6-month follow-up period through medical records and were incorporated into the prespecified composite primary outcome. No additional adverse events were systematically recorded.

16a. How sample size was determined

The sample size was determined based on the primary composite outcome of change in general self-efficacy and rehospitalisation or death at 6 months. To achieve 80% power at a two-sided significance level of 0.05 to detect an increase in the proportion of improved patients from 20% in the control group to 40% in the intervention group, a minimum of 91 participants per group was required. To allow for withdrawals and loss to follow-up, the planned sample size was increased to 110 participants per group.

16b. Interim analyses and stopping criteria

No interim analyses were planned or conducted, and no formal stopping guidelines were specified in the study protocol.

17a. Sequence Generation

The random allocation sequence was generated by an independent third party using a computer-generated randomisation list.

17b. Type of Randomisation

Participants were randomised in a 1:1 ratio to intervention or control. Randomisation was stratified by age (<65 or ≥65 years) and diagnostic group (COPD, CHF, or both).

18. Allocation concealment mechanism

The allocation sequence was generated by an independent third party and was concealed from the enrolling healthcare professionals until participants had provided written informed consent and were assigned to study groups.

19. Implementation

Designated healthcare professionals screened medical records, contacted eligible patients, and obtained written informed consent. After consent was received, participants were assigned to intervention or control according to the pre-generated allocation sequence. The randomisation list was generated by an independent third party and was not accessible to the enrolling healthcare professionals prior to assignment.

20a. Who was blinded

Due to the nature of the intervention, neither participants nor healthcare professionals delivering the intervention were blinded to group allocation. Outcomes included self-reported measures and register-based events (hospitalisation and death). No formal blinding of outcome assessors or data analysts was specified in the study protocol.

21a. Comparing groups

In the original PROTECT trial, between-group differences were calculated using the Pearson chi-square test for categorical variables, the Fisher exact test for dichotomous variables, and the independent 2-tailed Student t test for continuous variables. Between-group differences in the composite score were tested using the Fisher exact test for the dichotomous version and the Mantel-Haenszel chi-square test for the ordered categorical version. Binary logistic regression was used to calculate odds ratios with 95% CIs for the dichotomous version of the composite score. The Student t test was used to compare the mean change in GSE scores between groups. Between-group differences in improvement of ≥5 points on the GSE scale were calculated in the same way as the dichotomous version of the composite score. Bivariate correlations were computed using Pearson r. Missing outcome data for the 3- and 6-month follow-ups were imputed using the last value carried forward. Sensitivity analyses were conducted to assess robustness. Both intention-to-treat (ITT) and per-protocol (PP) analyses were conducted. The PP group included participants with at least one PCC phone call and at least one health plan who logged into the platform and used at least one of its functions. The significance level was set at P<.05 (2-sided).

Harms, including hospitalisations and deaths, were monitored and compared descriptively between groups as part of the composite outcome.

21b. Definition of who is included in each analysis

The primary analysis of the original PROTECT trial was conducted according to the intention-to-treat principle and included all randomised participants, analysed in the groups to which they were allocated.

A per-protocol analysis was also prespecified, including participants in the intervention group who received at least one person-centred telephone contact, had a personal health plan, and used at least one function of the digital platform.

21c. Missing Data

In the original PROTECT trial, missing outcome data at follow-up were handled using last observation carried forward. Sensitivity analyses were conducted to assess the robustness of the findings to missing data.

21d. Additional Analyses

In the original PROTECT trial, both intention-to-treat and per-protocol analyses were prespecified. Prespecified subgroup analyses were planned, and sensitivity analyses were conducted to assess the robustness of the findings, including analyses related to handling of missing data and assumptions in secondary and health economic analyses.

22a. Participant Numbers

A total of 224 participants were randomised, of whom 2 withdrew consent before analysis, leaving 222 participants (112 allocated to usual care and 110 to person-centred care).

All randomised participants were included in the intention-to-treat analysis and analysed according to their allocated group.

A per-protocol analysis included 76 participants in the intervention group who fulfilled predefined criteria for intervention use.

22b. Losses and exclusions

Of the 224 participants randomised, two withdrew consent prior to analysis, leaving 222 participants (112 in the control group and 110 in the intervention group) included in the intention-to-treat analysis.

At the 3-month follow-up, questionnaire data were missing for 15 participants (11 in the intervention group and 4 in the control group). At 6 months, 19 participants had missing questionnaire data (13 in the intervention group and 6 in the control group). Missing outcome data were handled using last observation carried forward.

23a. Dates

Participants were recruited between August 2017 and June 2019. The intervention period lasted 6 months for each participant. The primary outcome was assessed 6 months after randomisation, with additional follow-up for secondary outcomes extending to 12 and 24 months. Follow-up for hospitalisation and death was conducted during the 6-month intervention period.

23b. Reasons for stopping recruitment

Recruitment continued until the planned sample size was reached. The trial was not stopped early and no interim stopping guidelines were applied. The study ended as planned after completion of the predefined recruitment period and follow-up.

24a. As administered

The intervention was delivered by five designated healthcare professionals (three registered nurses, one occupational therapist, and one physiotherapist) trained in person-centred care (PCC). Participants received structured telephone support combined with access to a digital platform over 6 months. The number of telephone calls was tailored to individual needs; the median number of calls was 4 (range 0–11).

A per-protocol group was defined as participants who received at least one PCC phone call, had a health plan established, logged into the platform, and used at least one of its functions.

Intervention fidelity was supported through regular meetings, training in PCC communication, peer review of telephone calls and health plans, and monitoring by researchers and patient representatives.

24b. Concomitant Care

Participants in both groups received usual care according to current clinical guidelines. No restrictions were imposed on concomitant medical treatment. Medication adjustments and other clinical management were performed at the discretion of the treating physician.

The control group had no follow-up telephone support.

25. Baseline Data

Baseline demographic and clinical characteristics for each group are presented in Table 1 of the original trial publication (14). The treatment and control groups were comparable at baseline, with no clinically meaningful differences in demographic or disease-related characteristics.

26. Numbers analysed, outcomes, and estimation

All randomised participants (n=222) were included in the intention-to-treat analyses. Outcome data at 3 and 6 months were available for the majority of participants, with missing data handled using last observation carried forward.

Results for the primary composite outcome and secondary outcomes are presented in Tables 2 and 3 of the original trial publication (14). Effect estimates are reported as odds ratios with 95% confidence intervals for binary outcomes and mean differences for continuous outcomes. Both absolute group proportions and relative effect measures are provided.

27. Harms

During the 6-month intervention period, four hospitalisations were recorded (three in the intervention group and one in the control group). No deaths occurred during the study period.

No other adverse events related to the intervention were reported. The trial was not stopped early for safety reasons.

28. Ancillary Analyses

In addition to the primary intention-to-treat analysis, a per-protocol analysis was pre-specified and conducted to explore outcomes among participants who actively used the intervention. Sensitivity analyses were performed to assess the robustness of findings, including analyses using last observation carried forward for missing data. Subgroup analyses were conducted according to diagnostic group (COPD, CHF, or both).

Health economic and process evaluations were also pre-specified components of the trial.

The present refill adherence study represents a post hoc register-linked analysis of the original trial cohort.

29. Interpretation
The original PROTECT trial found no significant difference between groups in the primary intention-to-treat analysis of the composite outcome. However, a short-term improvement in self-efficacy was observed in the per-protocol analysis at 3 months. No sustained effect was demonstrated at 6 months.

The findings were interpreted in light of the relatively stable baseline status of participants, possible ceiling effects in self-efficacy, and the timing and intensity of the intervention. The intervention was considered low risk, with few hospitalisations and no deaths observed.

Results were discussed in relation to previous studies suggesting that person-centred care may improve self-efficacy, particularly among patients with greater need or more severe disease.

30. Limitations

The trial had several limitations. First, the study population had relatively stable disease and high baseline self-efficacy scores, limiting the potential for measurable improvement and introducing possible ceiling effects. Second, the low number of hospitalisations reduced statistical power to detect differences in this component of the composite outcome.

The open-label design may have introduced performance bias, although blinding was not feasible given the nature of the intervention. The per-protocol analysis, while informative, should be interpreted cautiously as it may introduce selection bias.

Finally, the intervention was delivered within a specific primary care setting, which may limit generalisability to other healthcare contexts.
