# Supplementary material for: Remote person-centred care and long-term medication management in primary care: post hoc analysis of a randomised controlled trial
Source: BMJ Open. 2026 Jul 1;16(7):e118720. doi: 10.1136/bmjopen-2026-118720 (PMC13331147; doi:10.1136/bmjopen-2026-118720)
Supplement: Supplementary file 5 [file bmjopen-2026-118720.reviewer_comments.pdf]

## PEER REVIEW HISTORY

BMJ Open publishes all reviews undertaken for accepted manuscripts. Reviewers are asked to complete a checklist review form and are provided with free text boxes to elaborate on their assessment. These free text comments are reproduced below.

### ARTICLE DETAILS

**Title (Provisional)**

Remote person-centred care and long-term medication management in primary care: post-hoc analysis of a randomised controlled trial

**Authors**

Mannheimer, Stina; Fors, Andreas; Holst, Anna; Gyllensten, Hanna

---

### VERSION 1 - REVIEW

---

| | |
|---|---|
| **Reviewer** | **1** |
| **Name** | **Schmiemann, Guido** |
| **Affiliation** | **Universität Bremen, Health Services Research** |
| **Date** | **08-Apr-2026** |
| **COI** | **None** |

---

Thank you for inviting me to review this secondary analysis of the RCT "Protect" on patient-centred care (PCC).

The article is well written, structured and easy to follow.

But my major concern is the lack of a sound hypothesis. The authors describe their primary aim to „study the impact of the remote PCC intervention [..] on refill adherence to prescribed treatment“.

However, a convincing hypothesis or a model on how and why an intervention which does not address medication management in a standardised manner should have an impact on medication adherence remains unclear.

It appears that the analysis was not planned in advance, i.e., it was not mentioned in the study protocol. Furthermore, the results of the main study did not indicate any problems regarding the extent of medication adherence.

According to the results of the main trial, only short-term improvement (at 3 months, not at 6 months) in patients' perceptions of self-efficacy, lower societal costs and QALY were observed.

These results are interesting and may warrant more analysis. However, how medication adherence contributes to this remains unclear.

Further aspects:

1) For the description of PCC, it would be interesting if the support was given by the same team member during the study period and if the frequency of the contacts had an influence on the results.

2) Regarding the medication analysis: Why were diuretics and digitoxin not included in the medication analysis, as both are frequently used for CHF?

3) What about medications that were stopped or reduced during the trial? Were they counted as non-adherent?

---

| | |
|---|---|
| **Reviewer** | **2** |
| **Name** | **Symanowski, James** |
| **Affiliation** | **Levine Cancer Institute, Department of Cancer Biostatistics** |
| **Date** | **21-Apr-2026** |
| **COI** | **None** |

---

The current study involves analyzing Participant level data from a previously conducted clinical trial (PROTECT) linked with patient level data from a national registry.

Suggested comments to improve the manuscript follow.

1. An explanation for how participant level data from PROTECT were link to individuals from the Swedish Prescribed Drug Register should be provided. Typically, data exported from a clinical trial are in the form of a Limited Dataset, that includes a Study ID code to index the participant. Additionally, data from national registries are typically de-identified. The manuscript should be revised to explain how these individual patient level linkages were achieved and how PHI exposure to the analysis team were not compromised.

2. Although you provide inclusion criteria for this post-hoc analysis, a brief explanation for how the analysis population reduced from n=222 to n=99. It is acknowledged that these exclusions are details in Figure 1. However, a brief descriptive summary of these exclusions should be provided in the main text. Additionally, explain in this brief summary statement that the number of excluded patients were very well balanced between the study arms for each exclusion criterion.

---

## VERSION 1 - AUTHOR RESPONSE

<u>**Reviewer 1**</u>

***Comment**:* “Thank you for inviting me to review this secondary analysis of the RCT "Protect" on patient-centred care (PCC).
The article is well written, structured and easy to follow.
But my major concern is the lack of a sound hypothesis. The authors describe their primary aim to „study the impact of the remote PCC intervention [..] on refill adherence to prescribed treatment“.
However, a convincing hypothesis or a model on how and why an intervention which does not address medication management in a standardised manner should have an impact on medication adherence remains unclear.
It appears that the analysis was not planned in advance, i.e., it was not mentioned in the study protocol. Furthermore, the results of the main study did not indicate any problems regarding the extent of medication adherence.
According to the results of the main trial, only short-term improvement (at 3 months, not at 6 months) in patients' perceptions of self-efficacy, lower societal costs and QALY were observed.
These results are interesting and may warrant more analysis. However, how medication adherence contributes to this remains unclear.”

***Response***: We thank the reviewer for this important and thoughtful comment. We agree that the PROTECT intervention was not designed as a standardised medication-management intervention and that the present refill adherence analysis was not prespecified in the published study protocol. However, analyses of medication use over time were planned already in the original ethical application, including linkage to the Swedish Prescribed Drug Register (held by the National Board of Health and Welfare), with medication-related variables included for exploratory analyses. Our rationale was that PCC may plausibly influence medication-taking indirectly through strengthened patient–clinician partnership and increased attention to individual barriers and resources related to long-term condition management, although this mechanism was not explicitly targeted or formally tested in PROTECT. This rationale is also supported by previous research suggesting that nurse-led interventions and trustful patient–clinician relationships may contribute to improved medication adherence in chronic disease management. Previous studies investigating PCC and medication-related outcomes have often used more narrowly defined interventions specifically targeting medication management or prescribing practices. In contrast, we wanted to explore whether a broader and more clinically pragmatic PCC intervention,

reflecting routine primary care practice and centred on patients' narratives and priorities, could also be associated with medication-taking. We agree with the reviewer that the absence of an observed effect may reflect the non-standardised and indirect nature of the intervention. In response to the reviewer's comment, we have revised the manuscript to explicitly describe the study as an exploratory post-hoc analysis and softened causal wording regarding intervention "impact". We have also clarified the theoretical rationale for examining refill adherence as a potential indirect outcome of PCC. Relevant revisions were made in the *Abstract, Introduction, Materials and methods*, and *Strengths and limitations* sections.

***Further aspects:***

***1)*** "For the description of PCC, it would be interesting if the support was given by the same team member during the study period and if the frequency of the contacts had an influence on the results."

***Response***: The intervention was delivered by a team of five healthcare professionals trained in PCC. Participants often had repeated contact with the same healthcare professional, although support could also be provided by different members of the PCC team during the intervention period. We have clarified this in the *Intervention* section. The frequency of contacts was individualised according to participants' needs and preferences and was therefore not standardised within the intervention. We agree that contact frequency could potentially influence outcomes. However, given the limited sample size in the present post-hoc cohort, additional analyses of contact frequency and refill adherence were considered unlikely to provide robust or clinically informative results. In addition, previously published analyses from the full PROTECT trial population did not demonstrate associations between contact frequency and the main trial outcome. We have added this consideration to the *Strengths and limitations* section.

***2)*** "Regarding the medication analysis: Why were diuretics and digitoxin not included in the medication analysis, as both are frequently used for CHF?"

***Response***: Diuretics and digoxin were not included because the aim was to assess refill adherence for guideline-recommended long-term maintenance therapies for CHF and/or COPD with relatively stable dosing and clear refill expectations. Loop diuretics are

frequently adjusted according to symptoms and clinical review, making refill coverage difficult to interpret as non-adherence. Digoxin is not recommended as routine therapy for CHF in contemporary regional treatment recommendations and was therefore not included among the predefined medication categories analysed for refill adherence. We have clarified the medication selection principles in the *Methods* section.

***3)*** What about medications that were stopped or reduced during the trial? Were they counted as non-adherent?"

***Response***: We thank the reviewer for this important methodological question. Refill adherence was estimated from dispensing data in the Swedish Prescribed Drug Register by comparing the amount of medication dispensed with the expected consumption based on the prescribed daily dose. If the prescribed dose changed over time, the expected consumption was adjusted accordingly using the dosage text available at each dispensing. However, because the analysis was based solely on dispensing registry data and no review of medical records was performed, we could not determine whether a medication had been intentionally discontinued by the prescriber. The analysed medications mainly represented long-term maintenance therapies for CHF and/or COPD, for which continued treatment is generally expected over time. Nevertheless, absence of further dispensing could reflect either treatment discontinuation or non-adherence. We have clarified this limitation in the *Materials and methods* and *Discussion* sections.

**Reviewer 2**

***Comment 1:*** "An explanation for how participant level data from PROTECT were link to individuals from the Swedish Prescribed Drug Register should be provided.  Typically, data exported from a clinical trial are in the form of a Limited Dataset, that includes a Study ID code to index the participant.  Additionally, data from national registries are typically de-identified.  The manuscript should be revised to explain how these individual patient level linkages were achieved and how PHI exposure to the analysis team were not compromised."

***Response***: We have added information on the linkage procedure. In brief, participants in PROTECT provided informed consent for linkage to the Swedish Prescribed Drug Register and other national registers. Linkage was performed using the Swedish personal identity

number by authorised personnel in accordance with ethical approval and Swedish data-protection regulations. The clinical trial data were transferred to Statistics Sweden (SCB), where personal identity numbers were replaced with pseudonymised study-specific codes. The pseudonymisation key was securely retained by SCB and was not available to the research team. Registry holders, including the National Board of Health and Welfare, received the pseudonymised identifiers and returned registry data linked through these study-specific codes. Consequently, the analysis dataset available to the research team contained only pseudonymised study IDs and no directly identifiable personal information, while still allowing accurate individual-level linkage across datasets. We have clarified this in the *Materials and methods* section.

***Comment 2***: Although you provide inclusion criteria for this post-hoc analysis, a brief explanation for how the analysis population reduced from n=222 to n=99. It is acknowledged that these exclusions are details in Figure 1. However, a brief descriptive summary of these exclusions should be provided in the main text. Additionally, explain in this brief summary statement that the number of excluded patients were very well balanced between the study arms for each exclusion criterion.

***Response***: We thank the reviewer for this helpful comment and have clarified the exclusion process in the *Results* section. In brief, 123 of the 222 PROTECT participants were excluded mainly due to multidose dispensing, absence of dispensed CHF/COPD medication during follow-up, or dispensing records that did not allow calculation of daily dose. The exclusions were well balanced between the intervention and control groups, resulting in a final analysis population of 50 intervention and 49 control participants.

**We thank the reviewers for their constructive comments, which helped improve the clarity and interpretation of the manuscript.**

## VERSION 2 - REVIEW

| | |
|---|---|
| **Reviewer** | **1** |
| **Name** | **Schmiemann, Guido** |
| **Affiliation** | **Universität Bremen, Health Services Research** |

**Date** **02-Jun-2026**

**COI**

---

Thank you very much for your comprehensive and thorough response to my comments. The changes in the new manuscript fully address my concerns, and I agree to its publication in its current form.
